# Round the Hospitals

**Published:** 1920-05-29

**Authors:** 


### ROUND THE HOSPITALS.

The King and Queen took advantage of their visit to Aldershot to visit the Cambridge Hospital, where they were received by Lieut.-Colonel H. C. R. Hime, D.S.O., the Commandant, and Miss E. M. Lyde, O.B.E., R.R.C., the Matron. Previous to this visit, which gave immense pleasure to both patients and staff, the Royal party were conducted by Brigadier-General A. E. W. Harman, commanding the First Cavalry Brigade, through the quarters of his command, and inspected the apparatus whereby the cavalryman's life has become one of the healthiest in the country. Cooking-houses, dining-halls, kitchens, clubs and messes were visited, and the King and Queen showed their interest in all by many pertinent questions. It is seldom remembered that the Army owes the excellence of its

**ROUND THE HOSPITALS**—(*continued*).

domestic arrangements largely to the untiring efforts of Florence Nightingale, who was the first to awake in the War Office an understanding of the importance to the soldier of good food, cleanliness, fresh air, and recreation. Side by side with her untiring efforts for the sick, the pioneer of modern nursing carried on a ceaseless campaign, with a view to secure for the private soldier a better mode of life. She was able to show that the death-rate in the Army in time of peace from easily preventable causes was as though the authorities took 1,600 men out to Salisbury Plain every year and had them shot. The organisation which stirred the admiration of the King and Queen on Whit Monday is the fruit of her labours.

All nurses will learn with great regret that the severe bronchial attack from which Queen Alexandra has now happily recovered has slightly impaired her vision. Violent coughing is one of the distressing features of the complaint, and resulted in the bursting of a small blood-vessel in one of the Queen's eyes. There is every hope that the trouble will disperse, but Her Majesty is advised to rest and avoid exposure to weather. Queen Alexandra was not to be denied her usual reception of the nursing sisters who had received the decoration of the Royal Red Cross at the King's hands, but her engagements for some time to go must depend on the progress made towards complete recovery.

Princess Helena Victoria, G.B.E., President of the League of Mercy for the Home Counties, has accepted the presidency of the Reading Historical Pageant which is to take place in the Abbey ruins of that city from June 21 to 29. The Grand Stand affords accommodation for 2,000 spectators, and it is anticipated that a goodly sum will be realised for the funds of the League of Mercy, in aid of which the performance is being organised. There are to be 750 performers, drawn from all classes of the community, and so many momentous incidents have occurred in the past history of the place that the Rev. P. H. Ditchfield, the Pageant writer and deviser, has no lack of material to draw on. One entire episode will illustrate the old English song, "Sumer is y-cumen in," for this, the first extant piece of English musical counterpoint, and a very clever and delightful example, was the work of John of Forsete, a monk at Reading Abbey. The picture on the subject in this year's Royal Academy will be reproduced.

Sheffield is in the throes of an influenza epidemic, which is very bad news for the rest of the country. A spirited effort is being made to isolate patients, but unfortunately the City hospitals, where several empty wards are standing ready, are so poorly staffed that the nurses are physically incapable of coping with more sufferers. An appeal for volunteers with nursing experience has been circulated, and efforts are being made also to enlist the services of home helps for houses where the active workers are laid up. The Committee must be prepared to pay really well for aid in combating the disease, or they will not succeed in inducing a sufficient number of women to abandon their homes and previous work in order to expose themselves to infection. The usual rate of remuneration must be doubled at least if the need is to be met.

As recorded in our last issue, Queen Alexandra and the Duke of Connaught, who are respectively President and Grand Prior of the newly united British Red Cross Society and Order of St. John, have issued a powerful appeal for money and workers in order to carry out a strong programme. The post-war work which is urgently demanding attention falls under two categories—military and civil. For the first-named, the further care of the sick and wounded men of His Majesty's Forces, the Joint Council has sufficient funds at its disposal. For the other and wider sphere of activity, which includes the care of the tuberculous, financial aid to the voluntary civil hospitals, work parties for hospitals, child-welfare work, and assistance in all branches of nursing, health, and welfare work, the Council appeals for funds. It has set itself the heroic task of raising a million a year to relieve the necessities of the voluntary hospitals. The need for providing voluntary assistance for the nurses in hospitals inadequately staffed is very pressing, and hardly inferior in some quarters to the call for V.A.D. service during the war. We should like to see it the custom for girls to put in a year's service in hospitals on the completion of their school course, and are persuaded that this would in a great many cases set stirring a true vocation for the nurse's life.

The Indian Government appears to be seized with a fit of very ill-timed parsimony. The temporary nurses of the Q.A.M.N.S.I. are the only people who have not received a war gratuity. The refusal of the Government of India to grant these ladies gratuities has attracted much attention, and the Indian papers are full of indignant letters from officers and men they have nursed overseas. It is difficult to understand the parsimony of the authorities in this matter. The Madras Government has lately sanctioned an interesting experiment, no less than the training of ten Indian nurses in a general hospital. If successful the future of Indian nursing will be profoundly influenced by this step. The experience of the mission hospitals where native probationers are received goes to show that more than usual discrimination is needed as regards candidates and that a perceptibly higher average of failures than would be considered normal with Europeans must be accepted philosophically. It is the successes which count.

Retired members of Q.A.I.M.N.S. and demobilised members of Q.A.I.M.N.S. Reserve and of the Territorial Force Nursing Service are invited to attend a meeting to be held in the Grand Hall of St. Thomas's Hospital, Westminster, on Tuesday,

**ROUND THE HOSPITALS—(*continued*).**

June 1, at 3 P.M., to elect a representative to serve on the Sub-Committee of the United Services' Fund, as it is hoped to obtain a grant from that Fund to be used for the benefit of the members of these Services who have served during the war.

---

Dealing with the accounts of the West Riding County Council, the Government auditor states that in September 1918 arrangements were made for the sale throughout the county of dried milk for the use of nursing mothers and children. The milk is sold by the Council's nurses, who are supplied either direct by the dealer or through the divisional clerks, who collect all money received by the nurses. He found it extremely difficult and in some cases impossible to check the distribution and to verify the amount collected. No records have been kept by the nurses of the supplies received and distributed, and the stock-books kept by the divisional clerks were not kept in such a way as to show clearly at any time the amounts in stock held by the different nurses. He had suggested that each nurse should be required to keep a stock-book on the duplicating system, showing the quantities received, the issues, the cash received and the quantities remaining in store, one copy to be torn out and forwarded to the divisional clerk with each deposit of moneys received and the other to be retained in the book. The divisional clerks should open an account in their stock-books with each nurse showing the quantities supplied, etc. This matter is one of some importance as, he is informed, that up to date the purchases of dried milk have amounted to over £10,000 distributed by some 160 nurses. If the system of accounting suggested is properly carried out there ought to be no difficulty in checking the distribution and in ascertaining that all moneys to be collected have been duly received and accounted for.

---

There is a forecast in the Press that the Medical Consultative Council has recommended the Minister of Health to throw the onus of providing a nurse for every patient who is certified by a doctor to require one upon the local authorities. We await the issue of their full report, which will shortly be published, before commenting on this extraordinary suggestion. Meantime, there is a proverb about first catching your hare, which suggests itself unbidden. We have never ceased to regret that Dr. Addison could not see his way to adding a woman practically acquainted with nursing problems to the Consultative Council.

---

Nursing in Devonshire is still heavily handicapped by the scarcity of nurses. At the annual meeting of the Devon Nursing Association a great increase of desire for nursing aid was reported. Thirteen new branch Associations have affiliated during the year, bringing the total to 128, but everywhere the greatest difficulty is experienced in filling vacant posts. Some light is thrown on the causes of this scarcity in the Secretary's report. One cause can certainly not be deplored in the interests of sound nursing ideals. It is the inclination of women who are keen about nursing to take hospital training for three years, instead of qualifying only for village posts. Another cause which operates to deplete the county of nurses is the high marriage rate. Last year, with only ten nurses completing their training, the loss to the Association by the marriage of its village nurses was nine, while twelve other nurses gave up work in the county for other reasons. The County Council offers twenty scholarships of £50 each to candidates, who are not, however, forthcoming. Nurses' salaries have been raised to a minimum of £90, rising to £120, with allowance for uniform. It is not sufficient. A similar story was heard at the annual meeting of the South Wales Nursing Association—lack of money, lack of candidates.

---

We regret to learn of the death on April 27 of Miss Sophia Palmer, Editor-in-Chief of the *American Journal of Nursing*, which she organised from its inception and brought to a high pitch of popularity. She began her training as a nurse at the Massachusetts General Hospital in May 1876, before the introduction of aseptic surgery, worked for a time under Dr. Weir Mitchell and took a leading part in the organisation of training-schools for nurses in Massachusetts, Washington, and Rochester. She was the pioneer in the United States as regards State registration for nurses, and it is interesting to learn that it was first suggested to her on observing the wholesome effect of preparation for examination and registration on the young doctors in the hospital. She was the first President of the New York State Board of Nurse Examiners. With the notable exception of its almost grotesquely ill-informed foreign department, which unfortunately lay to some extent outside Miss Palmer's sphere, the *American Journal of Nursing* has earned a high reputation on both sides of the Atlantic for its wide grasp of the subjects on which it treats, and in particular for its admirable articles on nursing education and activities in the States.

---

A number of Medical Officers of Health met at Scarborough on May 14 to hear Dr. J. J. Buchan speak on the subject of the Health Visitor as a Public Health Officer. He spoke with great appreciation of the great work health visitors are doing, and incidentally exposed the need for general nursing training in the women who filled these posts; health visitation, he declared, should embrace all questions of personal health and hygiene, affecting all diseases, all classes and all ages of both sexes of the population. Where, we may ask, but in a general hospital for a term of three or four years can a knowledge be obtained of all diseases, at all ages, in both sexes? Dr. Buchan strongly deprecated the appointment of special health visitors for special diseases, as for tuberculosis, &c., and laid stress on the fact that the districts should be small.

**ROUND THE HOSPITALS**—*(continued)*.

The Committee of the Florence Nightingale Hospital has granted the matron three months' holiday to be taken in June, July, and August, including the month in which the hospital is closed. This is not because of illness, but as a recognition of the strenuous time she, like other matrons of civil hospitals, has had for the past five years in securing and keeping together a first-rate staff, and in bearing the burden of the constant worries from the ever-increasing costs and the growing work. This forethought of a committee of ladies might well be copied by other committees, thereby preventing many unnecessary breakdowns.

The annual report of King Edward's Coronation Fund for Nurses, Dublin, which was presented at the annual meeting on May 19, Sir Andrew Beattie, D.L., Trustee of the Society, presiding, reveals that during the past year the Fund has been able to extend a helping hand to eighteen nurses in distress through sickness, accident, old age, and other difficulties. Thanks to this timely help, these nurses have in many cases been saved from a permanent breakdown and been enabled to return to their work. Some of the cases helped by the Fund are those of nurses who at the age of fifty or sixty found themselves struggling with increasing ill-health and unable to compete with the younger generation. A pleasing feature of the work is that many of the subscribers to the Fund are nurses themselves, who have either been benefited by the Fund and give in gratitude, or who are glad to feel that the small sum they can contribute each year may mean one more fellow-nurse cheered and helped forward on her difficult way. During the year grants have been given to the extent of £119, apart from the Countess of Pembroke annuity, which brought £12 7s. to the holder. The expenses of administration did not amount to £20 and the balance in hand was £34 16s. 10d. That the Fund is administered in a kindly and practical fashion is evidenced by the alteration made unanimously to Rule 31, which provides that applicants for assistance must be recommended by two life governors of the Society. This proviso is being found increasingly difficult to carry out, as few of the life governors have personal acquaintance with applicants, and the word "referees" has now been substituted.

A pretty snapshot in the *Daily Mirror* of the crew from the London School of Medicine for Women training at Barnes for their coming race against Newnham College, brings to mind the value of this particular form of recreation for women engaged in medical and surgical work. We do not know of any boating club for nurses, but we feel confident that rowing will not long be neglected in institutions where the off-duty hours are sufficiently long to make it possible. It is a particularly invigorating exercise, demanding exposure to sun, wind, and weather, and banishing like a wet sponge over a slate the worries of a tired mind. As a novel and interesting experiment the boating club would seem to be marked out as the next step for some of the local centres of the College.

The advertisement in our columns last week of the post of Registrar to the General Nursing Council under the Nurses' Registration Act is of unusual interest. Candidates must be nurses not over fifty years of age holding a three years' certificate from a leading nurse-training school, and must, in addition, produce evidence of administrative ability and of being conversant with the standards of nursing education. A special form of application must be filled up and sent in before June 5. The salary offered is £550, which should secure a good choice of candidates for this highly interesting post. The department will be a large one, and its complete organisation from the beginning and control will devolve on the Registrar. It would be easy to use up all the fingers on both hands in ticking off the qualities which this lady *must* possess, but, if we had to choose one above all others, it would be the quality of fair-mindedness.

The Conference held in Brussels under the auspices of the Royal Institute of Public Health last week is of grave interest to nurses. On May 22 the subject was prophylaxis in connection with venereal disease, and a report was submitted from a special committee charged with investigations in Belgium and Great Britain. Several ladies, including Miss Norah March, spoke on the question, and the unanimous conclusion arrived at was that ignorance played a very large part in disseminating the disease. The figures quoted prove the widespread danger to the community. It is sufficient to note that of the individuals recognised by doctors as syphilitic one-third of the males and two-thirds of the females are entirely ignorant of the nature of the disease from which they are suffering or of its infective character.

The Council of the North Lonsdale Hospital, Barrow-in-Furness, is not content to sit still. It has again raised the salaries of the nursing staff, probationers receiving £20, £23 and £26, sisters £60 rising to £65, and the out-patient sister, who must possess the certificate of the Incorporated Society of Trained Masseuses for Massage and Electrical Treatment, £80. Indoor uniform is provided for all the staff. At present the probationers have a fifty-four hour week (sisters forty-eight hours). The next improvement the Hospital Council should take in hand is the reduction of the probationers' hours.

During her recent visit to Ireland Lady Aberdeen received the members of the Women's National Health Association at Larch Hill, her beautiful residence near Dublin. We understand that she has in view a great undertaking, no less than the establishment in this neighbourhood of an open-air home hospital for crippled and chronically invalided children. Such an institution is greatly needed in Ireland, and much interest has been aroused by the proposal.

